# Effect of dietary polyphenols along with exercise on hepatic transcriptional regulators of lipid metabolism

**DOI:** 10.3389/fnut.2025.1531327

**Published:** 2025-07-02

**Authors:** YiXiao Han, ZhiHao Chen

**Affiliations:** ^1^Henan University of Technology, Zhengzhou, China; ^2^College of Physical Education, Kunshan National University, Kunshan, Republic of Korea

**Keywords:** natural products, hepatic transcriptional factors, lipid metabolism, curcumin, resveratrol

## Abstract

The increasing dangers of aging dyslipidemia, such as cardiovascular disease, which is one of the major causes of mortality globally, must be prevented and managed due to the detrimental consequences of age-associated dyslipidemia, particularly its dangerous effects on the cardiovascular system. Numerous studies have shown that various forms of physical activity, including strength training and moderate aerobic workouts, have a good impact on middle-aged, adult, and elderly people’s lipid profiles, inflammatory responses, and cardiovascular risk. Besides exercise, natural substances found in fruits and plants, including polyphenols which have anti-inflammatory and antioxidant qualities, can also enhance health and slow down the effects of aging on changes in lipid profiles and cardiovascular irregularities. The impact of aging-related liver disease and dyslipidemia on cardiovascular abnormalities in the older population have been the subject of several research over the past 40 years. The precise mechanism of aging on the particular molecular mediators implicated in dyslipidemia in older adults has seldom been studied, despite the fact that several elements of the detrimental effects of aging on liver structure and function have been previously documented. Thus, in this study, we looked at how natural products and exercise affect hepatic transcriptional variables related to lipid metabolism.

## 1 Introduction

Polyphenols, a diverse group of naturally occurring compounds found abundantly in fruits, vegetables, tea, and red wine, have garnered significant attention for their potential health benefits, particularly in the context of metabolic disorders (Table 1) (1, 2). Among their myriad effects, polyphenols have been shown to play a crucial role in regulating lipid metabolism, primarily through their interactions with hepatic transcriptional factors. These compounds exert their influence by modulating the expression of genes involved in lipid synthesis, oxidation, and transport, ultimately contributing to the maintenance of lipid homeostasis (1). The liver is a central organ in lipid metabolism, orchestrating the balance between lipid storage and utilization. Transcriptional factors such as peroxisome proliferator-activated receptors (PPARs), sterol regulatory element-binding proteins (SREBPs), and liver X receptors (LXRs) are key regulators of these metabolic pathways. Polyphenols can interact with these transcriptional factors, either directly or indirectly, to enhance fatty acid oxidation, reduce lipogenesis, and improve overall lipid profiles (3–5).

**TABLE 1:** Classification, sources, biological activities, and health effects of major polyphenols.

Subclass	Representative compounds	Main dietary sources	Primary biological activities
Flavonols	Quercetin, kaempferol, myricetin	Onions, apples, kale, grapes	Antioxidant, anti-inflammatory, cardioprotective
Flavones	Luteolin, apigenin	Parsley, celery, peppers	Neuroprotective, anti-inflammatory
Isoflavones	Genistein, daidzein	Soybeans, legumes	Phytoestrogenic, metabolic regulation
Anthocyanins	Cyanidin, delphinidin, malvidin	Berries, red cabbage, grapes	Vascular protection, antioxidant
Flavanols	Catechins, theaflavins	Green tea, dark chocolate, apples	Vascular tone modulation, neuroprotection
Phenolic acids	Gallic acid, caffeic acid, ferulic acid	Tea, coffee, pomegranates, grains	Antioxidant, anti-inflammatory
Stilbenes	Resveratrol	Red wine, grapes, peanuts	Anti-aging, cardiometabolic regulation
Lignans	Secoisolariciresinol, matairesinol	Flaxseeds, grains, vegetables	Hormonal modulation, anti-proliferative
Tannins	Ellagitannins, proanthocyanidins	Wine, berries, nuts, tea	Antimicrobial, anti-inflammatory
Other (e.g., Baicalein)	Baicalein	Scutellaria baicalensis	Lipid-lowering, anti-inflammatory

Research has demonstrated that various classes of polyphenols, including flavonoids, phenolic acids, and stilbenes, can influence the activity of these transcriptional regulators. For instance, certain flavonoids have been shown to activate PPARs, promoting the expression of genes involved in fatty acid catabolism. Similarly, polyphenols may inhibit SREBP activity, leading to decreased lipid accumulation in hepatocytes. Through these mechanisms, polyphenols not only help mitigate the risk of conditions like non-alcoholic fatty liver disease (NAFLD) (6, 7) and dyslipidemia but also offer insights into dietary strategies for improving metabolic health. The effects of polyphenols on regulating lipid metabolism through hepatic transcriptional factors represent a promising area of research with significant implications for understanding the interplay between diet and metabolic health. As we delve deeper into these mechanisms, it becomes increasingly clear that incorporating polyphenol-rich foods into our diets may provide a valuable approach to managing lipid metabolism and preventing metabolic diseases.

## 2 Transcriptional control of hepatic lipid metabolism

Lipids serve various important functions in animals. They act as energy sources, form parts of cell membranes, and are precursors to various molecules involved in numerous biological processes, including steroid hormones, vitamins, bile acids, and eicosanoids. We obtain the lipids we need from our diet as well as through internal synthetic pathways. The regulation of glucose and lipid metabolism works together to manage energy expenditure and storage, keeping blood glucose levels within a tight range. Insulin and glucagon, produced by the pancreatic islets, play opposite roles in this regulatory process. After a period of fasting, reduced glucose levels trigger the release of glucagon from α cells in the islets, which primarily acts in the liver to boost glucose production by enhancing glycogen breakdown and gluconeogenesis (8, 9). In contrast, elevated glucose levels, such as those that occur after consuming carbohydrates, lead to the release of insulin from pancreatic β cells. This insulin facilitates the absorption and use of glucose and encourages the liver to synthesize glycogen and fatty acids. The fatty acids created through *de novo* lipogenesis (DNL), in addition to those absorbed from the bloodstream, are subsequently used to sequentially esterify glycerol to form triacylglycerols in the liver. DNL and fat production occur through a series of enzymes that are regulated to maintain metabolic balance in response to varying nutritional and hormonal environments (9, 10).

The process of synthesizing fatty acids and fats in the liver is a tightly controlled metabolic pathway essential for distributing energy. Lipogenic genes, which share similar characteristics in their promoter regions, are regulated together at the transcription level. Key transcription factors involved in this regulation include USF, SREBP-1c, LXR, and ChREBP. In this section, we would provide a summary of transcriptional regulation of hepatic lipid metabolism pathways.

### 2.1 SREBP transcription factors

Sterol regulatory element-binding proteins (SREBPs) are a group of transcription factors that manage lipid balance by regulating the expression of various enzymes essential for the production of cholesterol, FAs, TGs, and phospholipids. There are three isoforms of SREBP containing SREBP-1a, SREBP-1c, and SREBP-2 which each have distinct functions in lipid synthesis. SREBP-1a and 1c are generated from one gene called SREBF-1, which is found on human chromosome 17p11.2. In contrast, SREBP-2 comes from a different gene, SREBF-2, located on human chromosome 22q13. SREBPs are produced as precursor proteins that have two transmembrane helices, which help to hold the protein in the endoplasmic reticulum (ER) membrane. These proteins are linked with a cleavage-activating protein known as SCAP and an ER retention protein named Insig. For SREBP to become active, the SREBP-SCAP complex must separate from Insig, join with COPII-coated vesicles, and then move on to the Golgi apparatus. Within the Golgi, SREBPs undergo sequential cleavage by two proteases, S1P and S2P, leading to the release of the N-terminal fragment of the cytosolic section of the protein that translocates into the nucleus to function as the active transcription factor (11, 12).

Although SREBPs show some commonalities in activating target genes, various studies have revealed that different isoforms have specific functions. For instance, studies on mice shows that with an increased expression of nSREBP-1a in the liver, there is a significant rise in the activity of genes related to cholesterol production, such as HMG-CoA synthase, HMG-CoA reductase, and squalene synthase. This also occurs for fatty acid synthesis genes, including acetyl-CoA carboxylase (ACC), FA synthase (FAS), and stearoyl-CoA desaturase-1 (SCD-1), leading to an accumulation of cholesterol and TG (13, 14). Furthermore, mice that have high levels of hepatic nSREBP-1c show a specific increase in lipogenic gene expression, while there is no impact on genes involved in cholesterol production (15). On the other hand, the excessive production of the nSREBP-2 isoform in the livers of mice leads to a strong increase in the expression of genes related to cholesterol production, even though there is also a slight increase in the expression of genes associated with fatty acid synthesis (16). SREBP transcription factors are controlled through three main mechanisms: (1) transcription, (2) the proteolytic cleavage of their precursors, and (3) post-translational modifications of nSREBPs. While some regulatory mechanisms are shared among SREBP isoforms, there are notable distinctions. Specifically, it seems that SREBP-1a and SREBP2 are mainly regulated through precursor cleavage, whereas SREBP-1c appears to be primarily regulated at the transcriptional stage (17).

### 2.2 PPARα, PPARγ

Peroxisome proliferator activated receptors alpha and gamma (PPARα, PPARγ) are transcription factors that are activated by specific ligands and are part of the nuclear hormone receptor superfamily. The first member discovered in the PPAR family is PPARα, which is mainly found in tissues with high beta-oxidation rates, including the liver, kidney, heart, and muscle. Conversely, PPARγ is predominantly expressed in fat tissue. PPARs become activated through dietary fatty acids and eicosanoids. PPARα is crucial for the metabolism of fatty acids within cells. PPARα manages the expression of genes that encode for enzymes involved in the peroxisomal b-oxidation pathway, including ACO, and 3 ketoacyl-CoA thiolase. Furthermore, these enzymes are known to affect the metabolism of PPARα ligands. PPARα also regulates genes that participate in fatty acid uptake, conversion to acyl-CoA esters, mitochondrial b-oxidation, and the production of ketone bodies. Regulation of intracellular lipid metabolism is one of the many roles of PPARs. The levels of intracellular FAs are partially regulated by the fatty acid transport protein (FATP), which facilitates the intake of fatty acids across the cell membrane, and by acyl-CoA synthetase (ACS), which retains fatty acids in the cells by converting them into ester derivatives. Activation of PPARα leads to an increase in FATP expression in the liver and intestines, as well as heightened ACS expression in the liver and kidneys. The role of PPARα in fatty acid transport was additionally shown by the absence of increased levels of FATP and fatty acid translocase mRNA in the livers of PPARα-null mice when exposed to PPARα activators. Fatty acid metabolism is controlled by how quickly mitochondria take up fatty acids. PPARα has been shown to influence the import of fatty acids into mitochondria by increasing the expression of genes for muscle- and liver-type a-carnitine palmitoyltransferase I (18–20). Interestingly, the targeted blockade of mitochondrial fatty acid import in PPARα-null mice leads to the buildup of lipids in the liver and heart, resulting in hypoglycemia and death in all male subjects and 25% of female subjects. Additionally, PPARα-deficient mice that were given a high-fat diet exhibited substantial lipid accumulation in the liver (18, 21).

Other than this, *in vitro* and *in vivo* studies show that lipoprotein metabolism is also affected by PPARs. For instance, the initiation of lipoprotein lipolysis can occur due to either a rise in the natural activity of lipoprotein lipase or due to greater availability of triglyceride-rich lipoprotein particles for lipolysis, which is linked to a decrease in triglyceride-rich lipoprotein apo C-III levels (22). Furthermore, constraints on hepatic TG synthesis and very-low-density lipoprotein (VLDL) production arise from heightened FA uptake, increased FA breakdown, and decreased FA synthesis. An increase in the removal of LDL particles occurs due to alterations in the composition of plasma LDL, which subsequently enhances the affinity of LDL for its receptor and results in improved LDL breakdown (23, 24). Adipose tissue function is also another essential part of lipid metabolism system which is impacted by these transcriptional factors. White adipocytes primarily store energy as triglycerides, whereas brown adipocytes are designed to release energy as heat. The heat-generating capabilities of brown adipocytes are due to a specific protein known as UCP-1, which is a mitochondrial proton transporter that separates respiration from oxidative phosphorylation. PPARγ is highly expressed in both embryonic and adult brown adipose tissue (BAT). According to investigations, administering the PPARγ selective ligand rosiglitazone to CD-1 rats significantly increases the mass of interscapular BAT (25, 26). In laboratory studies, this ligand also promotes the final differentiation of the brown preadipocyte cell line HIB-1B and enhances the expression of UCP-1 along with various adipocyte-specific genes (27). In PPARγ is activated when preadipocytes transform into adipocytes and is prominently found in both white and brown fat tissue. When PPARγ, a nuclear receptor, is expressed in fibroblasts, it leads to the activation of genes specific to fat tissue and causes the cells to accumulate lipid droplets, which are characteristics typical of mature white adipocytes. Recent research has corroborated these initial findings, indicating that PPARγ mutants with dominant negative effects can impede adipogenesis (25, 28, 29).

### 2.3 Carbohydrate responsive element binding protein (ChREBP)

In 2001, K. Uyeda’s team used affinity chromatography and mass spectrometry to isolate a large protein consisting of 864 amino acids with a molecular weight of 94,600. This protein features multiple domains, such as a nuclear localization signal (NLS) located near the N-terminus, polyproline domains, a basic loop-helix-leucine-zipper (b/HLH/Zip), and a domain that is similar to a leucine zipper (Zip-like). This protein, known as ChREBP, was soon recognized as the long-desired glucose-responsive transcription factor (30). When ChREBP is overexpressed in primary cultures of hepatocytes, it triggers the activation of an L-pyruvate kinase (L-PK) reporter construct. ChREBP plays a crucial role in regulating the transcriptional response of glucose on the expression of glycolytic genes (like L-PK) and lipogenic genes (such as ACC and FAS) (30). Carbohydrates consumed in the diet are converted into acetyl CoA, an important compound in lipid metabolism. This acetyl CoA is generated by the oxidation of pyruvate, which is formed at the end of glycolysis, through the action of the enzyme pyruvate dehydrogenase (PDH) and fatty acid β-oxidation (30). It serves as a substrate for the synthesis of triglycerides and cholesterol, as well as for ketogenesis and the acetylation of proteins (31). When in the fed state, the elevated levels of nucleocytosolic acetyl CoA are efficiently used for lipid synthesis and the acetylation of histones. Conversely, during fasting or more intense situations, acetyl CoA is primarily sent to the mitochondria to enhance the production of adenosine triphosphate (ATP) and ketone bodies. Acetyl CoA is formed in the liver from glucose and fructose, which are quickly transformed into glyceraldehyde-3 phosphate (GAP) through glycolysis and fructolysis. GAP is transformed into pyruvate through the action of various glycolytic enzymes, including liver-type pyruvate kinase (encoded by Pklr), which is activated by ChREBP and facilitates the conversion of phosphoenolpyruvate to pyruvate. Subsequently, pyruvate is converted into acetyl CoA by the PDH complex located in the mitochondria (31, 32). Once acetyl CoA enters the tricarboxylic acid cycle, citrate production increases, and this citrate is transported out of the mitochondria, where it is converted back to acetyl CoA by ATP citrate lyase (encoded by Acly). Cytosolic acetyl CoA is transformed into long-chain fatty acyl CoA through the action of lipogenic enzymes, including acetyl CoA carboxylase 1 (encoded by Acc1) and fatty acid synthase (encoded by Fasn) (30–32).

### 2.4 Retinoid X receptor (RXR) and retinoic acid re94 ceptor (RAR)

Retinoid X receptors (RXRs) and retinoic acid receptors (RARs) are members of the nuclear receptor superfamily, which are critical for mediating the effects of retinoids, metabolites of vitamin A, in various physiological processes, including lipid metabolism. These receptors function as transcription factors that regulate gene expression in response to their ligands, thereby influencing a wide range of biological functions. RXRs are a class of nuclear receptors that can form heterodimers with other nuclear receptors, including liver X receptors (LXRs), peroxisome proliferator-activated receptors (PPARs), and thyroid hormone receptors. There are three isoforms of RXR: RXRα, RXRβ, and RXRγ, each encoded by distinct genes and exhibiting tissue-specific expression patterns (33–35). When activated by their ligands, such as 9-cis-retinoic acid, RXRs bind to specific response elements in target gene promoters, modulating transcriptional activity. In the context of lipid metabolism, RXRs play a pivotal role in regulating genes involved in fatty acid oxidation, lipogenesis, and cholesterol homeostasis. For example, RXR heterodimers with PPARs enhance the transcription of genes involved in fatty acid uptake and oxidation, thus promoting lipid catabolism in the liver (34–36).

Retinoic acid receptors, which include RARα, RARβ, and RARγ, are activated by all-trans-retinoic acid (the primary active metabolite of vitamin A) and play crucial roles in regulating cellular differentiation, growth, and metabolism. Similar to RXRs, RARs function as transcription factors that modulate gene expression upon ligand binding. In the liver, RARs influence lipid metabolism by regulating the expression of genes involved in fatty acid synthesis and degradation. RAR activation can enhance the transcription of enzymes involved in lipogenesis while simultaneously suppressing genes associated with fatty acid oxidation. The balance between these opposing effects is critical for maintaining lipid homeostasis in hepatocytes (37, 38). The interaction between RXRs and RARs is particularly important for coordinating lipid metabolism in the liver. The formation of RXR-RAR heterodimers allows for cross-talk between retinoid signaling pathways and other metabolic pathways mediated by different nuclear receptors. Both RXRs and RARs can promote lipogenic gene expression. For instance, RARs can enhance the expression of sterol regulatory element-binding protein 1c (SREBP-1c), a key transcription factor that stimulates lipogenesis. When RARs form heterodimers with RXRs, this interaction can further amplify lipogenic signaling pathways (37, 38). Conversely, RXR heterodimers with PPARs can activate genes associated with fatty acid oxidation, such as carnitine palmitoyltransferase 1 (CPT1), which is essential for transporting fatty acids into the mitochondria for β-oxidation. This interplay is critical for balancing energy expenditure and storage. RXRs also interact with LXRs to regulate cholesterol metabolism. LXRs are involved in promoting the expression of genes that facilitate cholesterol efflux and inhibit cholesterol synthesis. By forming heterodimers with LXRs, RXRs help maintain cholesterol homeostasis in the liver (37, 38).

### 2.5 Liver X receptor (LXR) and farnesoid X receptor (FXR)

Liver X Receptors are a group of nuclear receptors that function as transcription factors. There are two main isoforms of LXR: LXRα and LXRβ. LXRα is predominantly expressed in the liver, while LXRβ is more widely distributed across various tissues. LXRs are activated by oxysterols, which are oxygenated derivatives of cholesterol (39, 40). Once activated, LXRs bind to specific DNA sequences known as LXR response elements (LXREs) in the promoters of target genes, leading to the transcriptional regulation of several key proteins involved in lipid metabolism (39, 40). One of the primary roles of LXR is to promote cholesterol efflux and inhibit its synthesis. By activating genes such as ATP-binding cassette transporter A1 (ABCA1), LXR facilitates the transport of cholesterol from peripheral tissues back to the liver for excretion. Furthermore, LXR regulates genes involved in fatty acid synthesis, such as sterol regulatory element-binding protein 1c (SREBP-1c), enhancing lipogenesis in response to excess lipid availability. In addition to its role in cholesterol metabolism, LXR also influences inflammation and insulin sensitivity. By modulating the expression of inflammatory cytokines and adipokines, LXR helps maintain metabolic homeostasis and prevent the development of insulin resistance, which is often associated with metabolic disorders like obesity and type 2 diabetes (41–43).

Farnesoid X Receptor is another important nuclear receptor that primarily regulates bile acid homeostasis but also plays a significant role in lipid metabolism. FXR is activated by bile acids, particularly chenodeoxycholic acid and other farnesoid derivatives. Upon activation, FXR binds to FXR response elements (FXREs) in target gene promoters, influencing the expression of genes involved in bile acid synthesis, transport, and metabolism. FXR’s role in lipid metabolism is multifaceted (44, 45). It regulates the expression of fibroblast growth factor 19 (FGF19), a hormone that inhibits bile acid synthesis in the liver by downregulating CYP7A1, the rate-limiting enzyme in bile acid production. This feedback mechanism helps maintain bile acid levels within a physiological range. Moreover, FXR has been shown to modulate lipid metabolism by influencing the expression of genes involved in triglyceride and cholesterol metabolism. For instance, FXR activation promotes the expression of genes that enhance fatty acid oxidation and reduce triglyceride accumulation in hepatocytes. By regulating these pathways, FXR helps prevent hepatic steatosis (fatty liver disease), a condition often associated with metabolic syndrome (46).

While LXR and FXR have distinct roles in regulating lipid metabolism, they also exhibit complex interactions that contribute to overall metabolic homeostasis. For example, both receptors can influence each other’s pathways; LXR activation can enhance FXR signaling by promoting the expression of FGF19, while FXR can modulate LXR activity by affecting bile acid levels (47). Moreover, both receptors play critical roles in responding to dietary changes. In conditions of high-fat or high-cholesterol diets, LXR and FXR work together to balance lipid uptake and storage while promoting excretion pathways to prevent excessive accumulation of lipids (47). In summary, Liver X Receptor (LXR) and Farnesoid X Receptor (FXR) are vital regulators of lipid metabolism in the liver. By coordinating various metabolic pathways related to cholesterol, fatty acids, and bile acids, these receptors help maintain lipid homeostasis and prevent metabolic disorders. Understanding their functions and interactions offers potential therapeutic targets for treating conditions like NAFLD, dyslipidemia, and other metabolic syndromes.

## 3 Polyphenols: from pharmacokinetics to medical applications

Polyphenols are a diverse group of naturally occurring compounds found abundantly in plants (Table 1). Characterized by the presence of multiple phenolic groups (aromatic rings with hydroxyl groups), polyphenols are known for their antioxidant properties and potential health benefits. With over 8,000 different types identified, polyphenols play crucial roles in plant physiology, protecting them from environmental stressors, such as UV radiation, pathogens, and herbivores (5, 48). In humans, polyphenols have been linked to a variety of health benefits, including anti-inflammatory, antioxidant, anticancer, and cardiovascular protective effects. This manuscript aims to explore the classifications, sources, and applications of polyphenols. Polyphenols can be classified in several ways based on their chemical structure and the number of phenolic groups they contain. Broadly, polyphenols are categorized into two major groups: flavonoids and non-flavonoids. These groups are further subdivided based on their structural features (1, 49, 50).

### 3.1 Flavonoids

Flavonoids are the largest subclass of polyphenols, known for their wide distribution in the plant kingdom. They are characterized by a 15-carbon skeleton structure consisting of two aromatic rings (A and B) linked by a three-carbon bridge (C). There are several types of flavonoids, each with distinct chemical properties and biological effects:

Flavonols. These are commonly found in onions, kale, apples, and grapes. Examples include quercetin, kaempferol, and myricetin.

Flavones. Found in parsley, celery, and peppers, examples include luteolin and apigenin.

Isoflavones. These are mainly present in soybeans and legumes. Common isoflavones include genistein and daidzein.

Anthocyanins. These are responsible for the red, purple, and blue colors of many fruits and vegetables, such as berries, red cabbage, and grapes. Examples include cyanidin, delphinidin, and malvidin.

Flavanols. These include catechins and theaflavins found in green tea, dark chocolate, and certain fruits like apples and grapes (51–53).

### 3.2 Non-flavonoids

Non-flavonoids are another major class of polyphenols. This group includes a variety of compounds that differ significantly in their chemical structure from flavonoids. Important subclasses of non-flavonoid polyphenols include:

Phenolic acids. These are further divided into two main categories—hydroxybenzoic acids (such as gallic acid, found in tea, and ellagic acid, found in pomegranates) and hydroxycinnamic acids (such as caffeic acid and ferulic acid, found in coffee and whole grains) (51–53).

Stilbenes. Resveratrol is the most famous stilbene, found in red wine, grapes, and peanuts. It has been studied for its potential anti-aging and cardiovascular benefits.

Lignans. Found in seeds (especially flaxseeds), whole grains, and vegetables, lignans like secoisolariciresinol and matairesinol are thought to have antioxidant properties and may contribute to hormone regulation.

Tannins. These polyphenolic compounds are abundant in tea, wine, and certain fruits like pomegranates. Tannins have astringent properties and have been shown to have antibacterial and anticancer effects (51–53).

### 3.3 Sources of polyphenols

Polyphenols are widely distributed in the plant kingdom, with fruits, vegetables, grains, nuts, seeds, herbs, and beverages being the primary dietary sources. Here are some common foods rich in polyphenols:

(A) Fruits and berries: berries (e.g., blueberries, strawberries, blackberries, raspberries) are some of the richest sources of polyphenols, especially anthocyanins, flavonols, and ellagic acid. Apples contain flavonoids such as quercetin and epicatechins, which have antioxidant properties. Grapes and red wine are high in anthocyanins, resveratrol, and flavonoids. Citrus fruits (e.g., oranges, lemons) provide flavonoids such as hesperidin and narirutin (51–53).

(B) Vegetables and legumes: kale and spinach are rich in flavonoids, especially quercetin and kaempferol. Tomatoes contain lycopene, a type of carotenoid that also exhibits polyphenolic properties. Beans, particularly soybeans, are significant sources of isoflavones like genistein and daidzein. Onions are a good source of quercetin and sulfur-containing compounds.

(C) Nuts, seeds, and grains: Flaxseeds are a notable source of lignans, while walnuts and almonds are rich in polyphenols such as ellagic acid and flavonoids. Whole grains like oats, barley, and rye contain phenolic acids such as ferulic acid and caffeic acid (51–53).

(D) Beverages: tea (especially green tea) is one of the best-known sources of polyphenols, particularly atechins, such as epigallocatechin gallate (EGCG). Coffee contains chlorogenic acid, a type of hydroxycinnamic acid, and is a major source of polyphenols in many diets worldwide. Red wine contains resveratrol and various flavonoids, which have been associated with cardiovascular health benefits (51–53).

### 3.4 Health benefits of polyphenols

**FIGURE 1:**
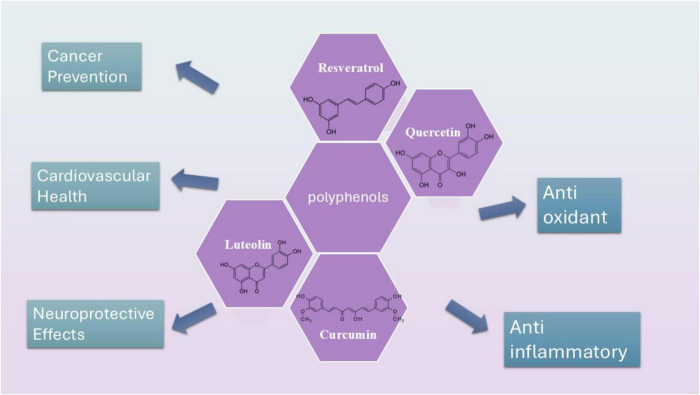
Schematic presentation of the most studied polyphenols including curcumin, resveratrol, luteolin, and quercetin and their structures.

Polyphenols have garnered significant attention due to their potential health benefits, primarily attributed to their antioxidant, anti-inflammatory, and antimicrobial properties (Figure 1). These compounds have been studied for their role in preventing and managing a variety of chronic diseases.

#### 3.4.1 Antioxidant activity

Polyphenols are potent antioxidants, capable of neutralizing free radicals that cause oxidative stress. Oxidative stress is linked to the development of chronic diseases like cancer, diabetes, and cardiovascular diseases. For instance, the high antioxidant activity of polyphenols like catechins in green tea, cardamom and resveratrol in red wine is thought to contribute to their protective effects against these diseases (54–57).

#### 3.4.2 Anti-inflammatory effects

Chronic inflammation is implicated in numerous health issues, including heart disease, arthritis, and neurodegenerative conditions. Polyphenols such as curcumin (from turmeric) and anthocyanins have been shown to suppress pro-inflammatory markers, thus potentially reducing the risk of inflammatory diseases (58, 59).

#### 3.4.3 Cardiovascular health

Polyphenols have been extensively researched for their cardiovascular benefits. For example, the flavonoids in dark chocolate and the resveratrol in red wine have been linked to improved endothelial function, reduced blood pressure, and a decreased risk of atherosclerosis. These effects are primarily attributed to their antioxidant and anti-inflammatory properties (60, 61).

#### 3.4.4 Cancer prevention

Several studies suggest that polyphenols, such as curcumin, resveratrol, and ellagic acid, may have anticancer properties. They can modulate various molecular pathways involved in cancer cell proliferation, apoptosis (programmed cell death), and metastasis. For example, resveratrol has been shown to inhibit cancer cell growth *in vitro* and in animal models, particularly in breast and colon cancer (60, 62).

#### 3.4.5 Neuroprotective effects

Emerging evidence suggests that polyphenols may play a protective role in brain health. Flavonoids, such as those found in berries, are thought to improve cognitive function and protect against age-related cognitive decline. Resveratrol and epigallocatechin gallate (EGCG) have also been shown to have potential in preventing neurodegenerative diseases like Alzheimer’s and Parkinson’s by reducing oxidative stress and inflammation in the brain (63, 64).

## 4 Dysregulation of lipid metabolism and its role in disease pathogenesis

Lipid metabolism is a complex biochemical process that involves the synthesis, storage, and degradation of lipids, which are crucial for cellular function, energy homeostasis, and the formation of cellular membranes. Proper regulation of lipid metabolism is essential for maintaining metabolic health. Dysregulation of this intricate network can lead to a variety of diseases, including obesity, type 2 diabetes, cardiovascular diseases, and NAFLD. This text aims to elucidate the mechanisms through which alterations in lipid metabolism contribute to the pathogenesis of these conditions (65–67).

### 4.1 Obesity

Obesity is characterized by excessive accumulation of adipose tissue and is often a consequence of an imbalance between energy intake and expenditure. Dysregulation of lipid metabolism plays a pivotal role in the development of obesity. Increased dietary intake of fats, coupled with reduced physical activity, leads to an overload of fatty acids in adipocytes (fat cells). This overload results in hypertrophy (enlargement) of adipocytes and promotes a state of chronic low-grade inflammation (68, 69). Inflammation in adipose tissue is mediated by the secretion of pro-inflammatory cytokines such as tumor necrosis factor-alpha (TNF-α) and interleukin-6 (IL-6), which can impair insulin signaling and promote insulin resistance. Insulin resistance is a hallmark of obesity and is closely linked to the development of type 2 diabetes. The dysregulated release of free fatty acids from enlarged adipocytes further exacerbates insulin resistance by interfering with glucose uptake in peripheral tissues (68, 69).

### 4.2 Type 2 diabetes mellitus

Type 2 diabetes mellitus (T2DM) is characterized by insulin resistance and impaired insulin secretion from pancreatic beta cells. Dysregulated lipid metabolism contributes significantly to the onset and progression of T2DM. In individuals with obesity, elevated levels of circulating free fatty acids can lead to lipotoxicity, damaging pancreatic beta cells and impairing their ability to secrete insulin effectively (70, 71). Moreover, excess lipids in the liver can lead to hepatic steatosis, a condition where fat accumulates in liver cells. This accumulation disrupts normal liver function and contributes to insulin resistance by altering hepatic glucose production and lipid synthesis. The interplay between dysregulated lipid metabolism and insulin resistance creates a vicious cycle that perpetuates hyperglycemia and exacerbates metabolic dysfunction (70, 71).

### 4.3 Cardiovascular diseases

Dyslipidemia is a major risk factor for cardiovascular diseases (CVD). It is often characterized by elevated low-density lipoprotein cholesterol (LDL-C), reduced high-density lipoprotein cholesterol (HDL-C), and increased triglyceride levels. Dysregulation of lipid metabolism can result from genetic factors, lifestyle choices, and underlying metabolic disorders (72–74). Elevated LDL-C levels contribute to the formation of atherosclerotic plaques in arterial walls, leading to atherosclerosis—a condition that narrows and hardens arteries. This process is driven by the infiltration of modified LDL particles into the arterial intima, triggering an inflammatory response that recruit’s macrophages and other immune cells. The accumulation of foam cells (macrophages laden with lipids) contributes to plaque formation and instability, increasing the risk of acute cardiovascular events such as myocardial infarction and stroke (72–74). Conversely, low levels of HDL-C are associated with impaired reverse cholesterol transport, a process through which excess cholesterol is removed from peripheral tissues and transported back to the liver for excretion. This dysfunction further exacerbates the risk of CVD by promoting cholesterol accumulation in arterial walls (72–74).

### 4.4 Non-alcoholic fatty liver disease (NAFLD)

Non-alcoholic fatty liver disease is characterized by excessive fat accumulation in liver cells in individuals who consume little or no alcohol. It ranges from simple steatosis to non-alcoholic steatohepatitis (NASH), which can progress to cirrhosis and hepatocellular carcinoma. Dysregulated lipid metabolism plays a central role in the pathogenesis of NAFLD. Increased *de novo* lipogenesis (the synthesis of fatty acids from non-lipid precursors) in the liver, often driven by insulin resistance and elevated levels of circulating free fatty acids, leads to excessive accumulation of triglycerides in hepatocytes. This accumulation triggers oxidative stress and inflammation, which contribute to liver cell injury and fibrosis. The interplay between lipid metabolism and other metabolic pathways, such as glucose metabolism and inflammation, underscores the complexity of NAFLD pathogenesis. For instance, inflammatory cytokines released from adipose tissue can further exacerbate liver inflammation and fibrosis (75–78).

## 5 How does exercise affect hepatic transcriptional regulators of lipid metabolism?

Exercise plays a significant role in modulating hepatic transcriptional regulators that govern lipid metabolism. Regular physical activity has been shown to influence the expression and activity of key transcription factors such as peroxisome proliferator-activated receptors (PPARα and PPARγ), sterol regulatory element-binding proteins (SREBPs), and carbohydrate-responsive element-binding protein (ChREBP) in the liver (79). We would summarize the most recent studies about the effects of exercise in regulating these transcriptional factors in this section.

In a study about SREBP-1, C57BL/6J mice were used to investigate the effects of exercise on the expression of lipogenic genes in skeletal muscle (80). Mice were housed under controlled conditions and provided unrestricted access to a standardized diet. Female mice began an exercise protocol involving forced swimming at 8 weeks of age, while control animals remained sedentary (80). After 2 weeks of swimming exercise, the expression of SREBP-1 and its downstream lipogenic genes—including acetyl-CoA carboxylase-1 (ACC-1), stearoyl-CoA desaturase-1 (SCD-1), and diacylglycerol acyltransferase-1 (DGAT-1)—was measured in the gastrocnemius and quadriceps muscles. Notably, mRNA levels were assessed either 3 or 22 h after the final exercise session. Prior research under similar conditions had shown increased GLUT4 mRNA expression post-exercise. The findings revealed that chronic exercise (e.g., 2 weeks of swimming or extended treadmill training), but not a single exercise session, significantly upregulated SREBP-1c and associated lipogenic genes in skeletal muscle. The discussion contextualizes these results within broader metabolic adaptations, noting that while excessive lipid accumulation in muscle is typically linked to insulin resistance in metabolic disease, trained athletes exhibit elevated muscle triglycerides alongside enhanced insulin sensitivity and oxidative capacity (80).

Another study aimed to investigate the effects of aerobic exercise on the expression of the SREBP-1c gene in the skeletal muscle of obese female rats (81). After 6 weeks of treadmill training, gene expression analysis of the quadriceps muscle revealed that the high-fat diet significantly suppressed SREBP-1c expression, while aerobic training reversed this effect, significantly increasing gene expression levels. These findings suggest that aerobic exercise can counteract the adverse effects of high-fat diets on skeletal muscle lipid metabolism by enhancing SREBP-1c expression, highlighting the therapeutic potential of regular aerobic activity in mitigating metabolic disturbances associated with obesity (81).

The study conducted by Nadeau et al. (82) explored the relationship between intramuscular triglyceride (IMTG) accumulation and the expression of SREBP-1 in animal models subjected to exercise training and long-term calorie restriction (CR). In a cohort of 38 Sprague-Dawley rats, exercise training led to elevated triglyceride levels in the gastrocnemius and soleus muscles. This increase was accompanied by higher mRNA and protein expression of SREBP-1c, including both its precursor and mature forms, as well as increased levels of fatty acid synthase (FAS) (82). Similarly, in rhesus monkeys (Macaca mulatta) subjected to an average of 10.4 years of caloric restriction, skeletal muscle biopsies showed elevated SREBP-1 protein levels and enhanced ERK1/ERK2 phosphorylation, suggesting increased anabolic signaling. Despite the rise in IMTG content, both exercise and CR were associated with improved insulin sensitivity. These findings indicate that SREBP-1 upregulation may play a central role in IMTG accumulation under metabolically healthy conditions, such as in trained athletes or calorie-restricted individuals, potentially supporting adaptive lipid storage without impairing insulin action (82).

Recent experimental findings support the role of exercise in modulating LXRα, a key transcriptional regulator involved in cholesterol homeostasis. In a controlled study using adult male Wistar rats, endurance training significantly increased hepatic LXRα mRNA expression along with an improvement in lipid profile (83, 84). Specifically, trained rats exhibited elevated HDL-C levels and reduced LDL-C, total cholesterol, and cholesterol ratios, while triglyceride concentrations remained unchanged. These results suggest that endurance exercise may facilitate reverse cholesterol transport via LXRα upregulation, contributing to the cardioprotective effects of physical activity. This mechanistic insight highlights the potential of exercise to influence transcriptional regulators of lipid metabolism in a manner complementary to polyphenol-based interventions (83, 84).

A group of adenosine triphosphate-binding cassette (ABC) transporters, including ABCA1, ABCG1, ABCG4, ABCG5, and ABCG8, facilitate cholesterol efflux from cells, making them key targets in the prevention and treatment of atherosclerosis (83). The ABCA1, ABCG5, and ABCG8 genes are activated by LXR and liver receptor homolog-1 (LRH-1), which are critical in the regulation of cholesterol metabolism. Oxysterols, derivatives of cholesterol containing additional oxygen functions such as hydroxyl, carbonyl, or epoxide groups, act as ligands for LXR, establishing LXR and LRH-1 as sensors of cellular cholesterol levels. LXRs are ligand-activated transcription factors that regulate genes involved in lipid, cholesterol, and bile acid metabolism, and their activity plays a significant role in atherosclerosis prevention. This study aimed to investigate the effects of 12 weeks of high-intensity interval training (HIT) and low-intensity continuous training (LIT) following a high-fat diet on LXRα gene expression in male Wistar rats (83). The results revealed significant differences in LXRα gene expression between groups (*P* ≤ 0.05), with the highest levels of LXRα gene expression observed in the HIT group and the lowest in the control group. The study demonstrated that 12 weeks of high-intensity interval training (HIT) and low-intensity continuous training (LIT) following a 13 weeks high-fat diet resulted in increased LXRα gene expression, which may serve as a predictive mechanism for atherosclerosis, particularly in individuals with obesity. Moreover, HIT training was found to be more effective in enhancing LXRα gene expression (83).

Another study aimed to explore whether endurance training enhances the expression of the liver LXRα gene (85). Twelve adult male Wistar rats (weighing 200–220 g) were divided into control and training groups. The training group underwent treadmill exercise at 28 m/min (0% grade) for 60 min per day, 5 days a week, for 8 weeks. Twenty-four hours after the final exercise session, the rats were euthanized, and blood samples were collected from the right ventricle. Plasma was analyzed for HDL-C, low-density lipoprotein cholesterol (LDL-C), total cholesterol (TC), and triglycerides (TG) (85). Additionally, liver tissue was excised, washed in ice-cold saline, and frozen in liquid nitrogen for the assessment of LXRα and ABCA1 mRNA levels. The data showed a significant increase in both LXRα and ABCA1 mRNA levels in the trained rats compared to the control group. Plasma HDL-C concentration was significantly higher (*P* < 0.001) in the trained rats by the end of the exercise program (85). Furthermore, LDL-C (*P* < 0.003), TG, TC concentrations, and the TC/HDL-C and LDL/HDL-C ratios were significantly lower in the trained rats compared to the controls (*P* < 0.001). In conclusion, endurance training led to a significant increase in LXRα gene expression, which was associated with elevated ABCA1 mRNA levels and plasma HDL-C concentration (85).

Peroxisome proliferator activated receptors alpha and PPARγ are transcription factors that are the most studies hepatic transcriptional factors in this field. A study began with a cohort of healthy participants who were assessed for baseline fitness levels and metabolic health markers (86). Participants engaged in a structured 8 weeks exercise regimen that included both aerobic and resistance training components, aimed at enhancing cardiovascular fitness and metabolic function. Plasma samples were analyzed for PPARγ activity before and after exercise sessions (86). Results indicated a statistically significant increase in PPARγ activity immediately following exercise at the beginning of the training program, suggesting that acute bouts of exercise can lead to an immediate increase in PPARγ ligands. In untrained individuals, there was a noticeable transient increase in the expression of PPARγ-regulated genes in monocytes after exercise. This increase was significant within hours of exercise but returned to baseline levels within 24 h, indicating a short-lived response to acute exercise (86). By the end of the 8 weeks training program, participants showed sustained increases in the expression of PPARγ target genes in monocytes, suggesting that regular exercise may lead to long-term adaptations in PPARγ signaling pathways. The study also explored correlations between changes in PPARγ activity and improvements in metabolic health indicators, such as insulin sensitivity and lipid profiles. Participants who exhibited greater increases in PPARγ activity tended to show more significant improvements in these metabolic parameters. Overall, the findings support the hypothesis that exercise enhances PPARγ activation in monocytes, which may contribute to the anti-inflammatory and antiatherogenic effects associated with regular physical activity, particularly beneficial for individuals at risk for metabolic disorders like type 2 diabetes. These results highlight the complex interplay between exercise, PPARγ signaling, and metabolic health, suggesting potential therapeutic avenues for enhancing health through physical activity (86).

Another study worked on PPAR-α and its role in regulating exercise-induced immune and metabolic responses in peritoneal macrophages (87). The research utilized C57BL/6 wild-type (WT) mice and PPAR-α knockout (KO) mice, assessing both groups in non-exercising control conditions (*n* = 4) and 24 h following acute moderate exercise (*n* = 8) (87). Key metabolic parameters, including glucose levels, non-esterified fatty acids, total cholesterol (TC), and triacylglycerol, were measured in serum samples. Additionally, cytokine concentrations (IL-1β, IL-6, IL-10, TNF-α, and MCP-1) were quantified from cultured peritoneal macrophages, with some samples stimulated using LPS (2.5 μg/mL) and Rosiglitazone (1 μM). Results indicated that exercised PPAR-α KO mice exhibited significantly lower glucose concentrations alongside elevated TC and TG levels in serum compared to their WT counterparts (87). At baseline, there were no notable differences in cytokine production between the two genotypes. However, following LPS stimulation, IL-1β levels were significantly elevated in KO mice. Both IL-6 and IL-1β concentrations remained higher in KO mice compared to WT mice, even after the exercise intervention. Notably, MCP-1 levels were not restored in the exercised KO group following LPS treatment. Furthermore, treatment with Rosiglitazone did not effectively reduce pro-inflammatory cytokine production in KO mice, whether at baseline or post-exercise (87). Importantly, acute exercise did not lead to changes in mRNA expression levels in WT mice. These findings suggest that PPAR-α plays a crucial role in maintaining glucose homeostasis and mediating anti-inflammatory responses associated with acute exercise. The absence of PPAR-α appears to result in an overexpression of pro-inflammatory cytokines in response to LPS stimulation. Additionally, neither moderate exercise nor PPAR-γ agonist treatment was able to mitigate this inflammatory response in the absence of PPAR-α. These results underscore the importance of PPAR-α in regulating metabolic and immune functions during exercise (87).

Another study aimed to examine the effects of an 8 weeks low-intensity exercise program on the expression of PPARγ co-activators (PGC-1α and PGC-1β), Th1 and Th2 cytokines, and macrophage polarization markers in sedentary individuals (88). Results demonstrated that the low-intensity exercise program led to a significant upregulation of M2 macrophage markers, as well as increased expression of PGC-1α and PGC-1β. Concurrently, there was a downregulation of M1 macrophage markers (88). Plasma levels of Th2 cytokines (such as IL-4 and IL-10) rose following the exercise intervention, while Th1 cytokine levels (notably IL-6) decreased. Importantly, other PPARs, specifically PPARα and PPARβ/δ, did not show significant changes in response to the exercise regimen. The findings suggest that participation in low-intensity exercise may promote the differentiation of monocytes toward an M2 macrophage phenotype via the activation of PPARγ and the co-activators PGC-1α and PGC-1β (88).

A recent systematic review is also conducted from January 2000 to May 2022. Results indicated that 14 of the studies found that various forms of exercise programs, such as regular exercise, resistance training, swimming, climbing, and treadmill running, significantly improved PPAR-γ levels in individuals with T2DM, obese populations, and healthy subjects. Only two studies did not report a significant enhancement in PPAR-γ levels following physical activity (89). Importantly, all studies involving T2DM subjects demonstrated improved PPAR-γ levels after engaging in exercise training. Conclusions drawn from this review suggest that different types of aerobic exercise, irrespective of their specific nature and duration, can effectively up-regulate PPAR-γ messenger ribonucleic acid (mRNA) expression. This research provides a foundational basis for further investigations into the relationship between various forms of exercise training and PPAR-γ levels and activity across diverse human populations. However, due to the limited number of human studies available, there is a clear need for additional high-quality research to establish more definitive conclusions (89).

Taken together, exercise training in different types and forms has the capacity to regulate the mentioned transcriptional factors and thereby, affect lipid metabolism. The question the remains is that either this effects can be increased when combined with polyphenols or not.

## 6 Polyphenols and lipid metabolism: what is the correlation?

Polyphenols influence a wide range of cellular and molecular pathways, including those involved in lipid metabolism, primarily through modulation of hepatic transcriptional regulators. Emerging evidence suggests that these effects may be further enhanced when polyphenol intake is combined with physical exercise, due to overlapping targets such as PPARs and SREBPs. In this section, we summarize the existing literature on how polyphenols, exercise, and their combination influence key transcription factors involved in lipid synthesis, oxidation, and transport, highlighting both consistent findings and areas of variability.

### 6.1 SREBP

#### 6.1.1 Curcumin

**FIGURE 2:**
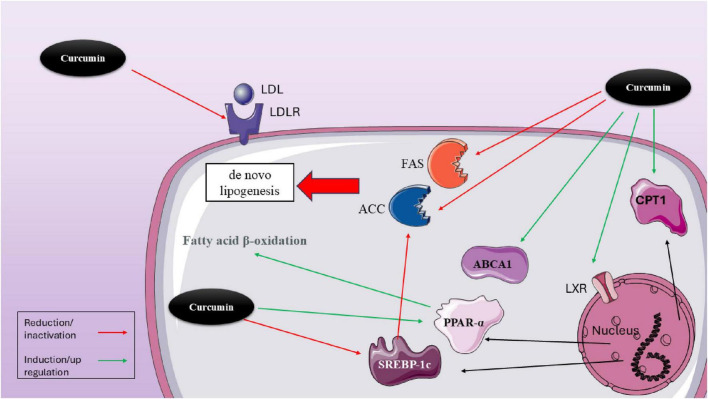
Molecular mechanisms of curcumin in modulating lipid metabolism via hepatic transcriptional regulators. This figure illustrates the multifaceted effects of curcumin on lipid metabolism within hepatocytes. Curcumin modulates several transcriptional regulators and enzymes that influence lipid synthesis, uptake, and oxidation. Notably, curcumin reduces LDL receptor (LDLR) expression, which can limit LDL uptake. It also downregulates lipogenic enzymes, including acetyl-CoA carboxylase (ACC), fatty acid synthase (FAS), and SREBP-1c, thereby inhibiting *de novo* lipogenesis. In contrast, curcumin activates lipid oxidation pathways by upregulating PPARα, carnitine palmitoyltransferase 1 (CPT1), and ABCA1, supporting fatty acid β-oxidation and cholesterol efflux. Through activation of LXR, curcumin indirectly influences the expression of ABCA1 and other lipid-related genes. The combined effects of curcumin demonstrate a net shift from lipid accumulation toward enhanced lipid utilization and export, offering therapeutic potential in metabolic disorders such as NAFLD and dyslipidemia.

Curcumin (CUR) is one of the most studied polyphenols in this field (all studies are summarized in Table 2). There are some *in vitro* studies which have confirmed the impact of CUR on SREBP expression. One of the first studies was conducted by Kang and Chen which examined CUR on hepatic satellite cells and observed that CUR decreased the levels of LDL receptors (LDLR) in these cells, leading to a reduction in cellular cholesterol levels. This effect was possible through regulating the levels of SREBP. However, they declared that CUR affects this transcriptional factor through the activation of PPARγ (90). Yuan et al. (91) gathered 23 apoE−/− mice and categorized them into three groups: control (*n* = 7), curcumin-treated (*n* = 8), and Lovastatin-treated (*n* = 8). They fed these mice with 20 mg/kg/day for 4 months and examined Intracellular lipid level, Serum lipid and lipoprotein, and atherosclerotic lesions in these mice. Eventually, they observed that curcumin is able to decrease the size of atherosclerotic plaques, stabilize them, decrease TC, TG, and LDL-C serum levels, while increasing HDL, and decrease cholesterol accumulation. It seems that one of the mechanisms by which CUR exerts all these effects is through inhibiting high fat-induced e SREBP-1 expression in apoE−/− mice and inhibiting the SREBP-1/caveolin-1 pathway (91). Immunofluorescence examinations also showed that curcumin treatment is able to inhibit the translocation of SREBP-1 from the cytoplasm into the nucleus (91). There are plenty of other *in vivo* studies that has observed the same results. For instance, according to a study, 100 mg/kg/day of curcumin for 8 weeks can significantly reduce both plasma and renal triglyceride levels in humans (92). Moreover, in diabetic rats treated with curcumin, there was an increase in AMPK phosphorylation. This treatment also inhibited the elevated renal expression of SREBP-1c, leading to a reduction in the levels of acetyl CoA carboxylase, fatty acid synthase, and adipose differentiation-related protein, which is an indicator of cytoplasmic droplets. These findings showed that curcumin helps prevent the progression of diabetic nephropathy via the AMPK–SREBP pathway (92). However, there are also some paradoxical results. A study shows that curcumin’s effects on SREBP expression are not directly exerted (93). In this study, Caco-2 cells were gathered to examine the dynamics of precursor and mature SREBP-2, SP-1, and SCAP in relation to dosage and timing. Following treatment with curcumin, researchers observed the distribution of SREBP-2 within the cells and assessed the expression of the S1P protein. Curcumin was found to lower the mRNA levels of SREBP2, SP-1, and SCAP, but it did not reduce the expression levels of precursor SREBP-2 (pSREBP-2) and SCAP at the same time. Additionally, curcumin appears to hinder the proteolytic processing of SREBP-2, leading to decreased production of the mature SREBP-2 (mSREBP-2) and altering the cellular distribution of SREBP-2 (93). The inhibitory impact of curcumin on SP-1 protein levels is temporary. While curcumin can decrease the mRNA and protein levels of S1P, it does not significantly affect the mRNA and protein levels of S2P (site-2 protease). Additionally, curcumin inhibits the proteolytic process of SREBP-2, leading to a reduction in mSREBP-2, which acts as a transcription factor that regulates genes involved in cholesterol metabolism. However, curcumin does not directly inhibit the expression of the mSREBP-2 protein (93). A similar study also indicated that CUR decreases the gene expression of SP-1 and lowers its trans-activation function, a process facilitated by the activation of PPARγ. Chromatin immuno-precipitation analysis confirms curcumin’s inhibitory impact on SP-1’s binding to the GC-box. In conclusion, their findings show that curcumin inhibits srebp-2 expression in cultured HSCs by activating PPARγ and diminishing SP-1 activity, which results in the downregulation of ldlr expression (94) (Figure 2).

**TABLE 2:** Studies exploring the effects of either polyphenols, exercise, or their combination on hepatic transcriptional factor, sterol regulatory element-binding protein (SREBP).

Polyphenol	Model of study	Method	Result(s)	References
Curcumin	*In vivo*	Control = 7, curcumin group = 8, lovastatin group = 8	Decreasing SREBP-1 expression and its translocation into the nucleus	(91)
	*In vivo*	6 weeks hamsters treated with curcumin for 12 weeks	Decreasing total cholesterol, TG and LDL and liver fat accumulation and hepatic steatosis, through reduced intestinal and hepatic SREBP-2 expression (*P* < 0.05)	(123)
	*In vivo*	Male Sprague–Dawley rats: control, diabetic and diabetic treated with curcumin (100 mg/kg/day) by gavage for 8 weeks	Decreasing plasma and kidney TG and increasing the phosphorylation of AMPK and prevented the increased renal expression of SREBP-1c and, as a result, decreased the expression of acetyl CoA carboxylase and fatty acid synthase	(92)
	*In vitro*	Caco-2 cells	Decreasing the proteolytic processing of SREBP-2, leading to decreased production and cellular distribution of the mature SREBP-2	(93)
	*In vitro*	Hepatic satellite cells	Dysregulating SREBP through the activation of PPARγ	(90)
	*In vitro*	Hepatic satellite cells	Inhibiting srebp-2 expression by activating PPARγ and diminishing SP-1 activity	(94)
	*In vivo*	Control groups: either distilled water or 5 g/kg thanol for 8 days. Treatment groups: either 300 mg/kg milk thistle or CUR before receiving ethanol	Increasing accumulation of hepatic lipid droplets caused by ethanol through decreasing SREBP-1c	(108)
	*In vitro*	HepG2	Increasing the expression of regulatory SREBP genes	(113)
Quercetin	*In vitro*	HepG2 cells	Increasing the expression of SR-BI in a concentration- and time-dependent manner	(96)
Baicalein	*In vitro*	HepG2 cells	Decreasing SREBP1	(97)
Resveratrol	*In vitro*	HepG2 cells	Reducing intracellular lipid droplets and decreasing hepatic steatosis and intracellular triglycerides through decreasing SREBP-1c	(124)

There the combination of CUR with aerobic training is able to reduce insulin resistance, lower serum insulin levels, decrease the ratio of low-density lipoprotein to high-density lipoprotein, and reduce the total cholesterol to high-density lipoprotein ratio, while increasing serum high-density lipoprotein cholesterol (95).

#### 6.1.2 Quercetin

**FIGURE 3:**
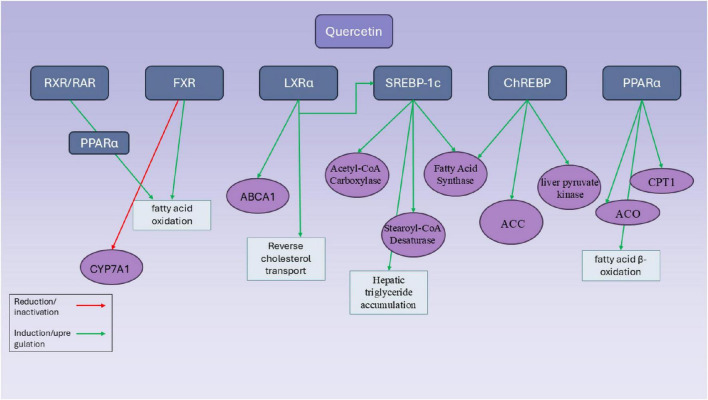
Mechanistic pathways of quercetin in modulating hepatic transcriptional regulators involved in lipid metabolism. This diagram illustrates the molecular targets and regulatory effects of quercetin on key hepatic transcription factors and enzymes involved in lipid metabolism. Quercetin influences multiple transcriptional regulators, including RXR/RAR, FXR, LXRα, SREBP-1c, ChREBP, and PPARα, through either induction (green arrows) or inhibition (red arrows). Notably, quercetin enhances fatty acid β-oxidation by upregulating PPARα and its downstream targets such as carnitine palmitoyltransferase 1 (CPT1) and acyl-CoA oxidase (ACO). It also promotes reverse cholesterol transport via LXRα activation and increased expression of ATP-binding cassette transporter A1 (ABCA1). Simultaneously, quercetin modulates lipogenesis by upregulating SREBP-1c and its downstream targets—acetyl-CoA carboxylase, fatty acid synthase, and stearoyl-CoA desaturase—leading to hepatic triglyceride accumulation. Furthermore, quercetin induces ChREBP activity and related genes such as liver pyruvate kinase and ACC, reinforcing its impact on glycolysis and lipogenesis. FXR activity is increased, promoting fatty acid oxidation, while CYP7A1 is suppressed via FXR and RXR-mediated pathways. Overall, quercetin exerts complex and sometimes paradoxical effects on hepatic lipid metabolism through a network of overlapping transcriptional controls.

Quercetin is also another member of the flavonoid family which is effective on regulating the expression of SREBPs *in vivo* and *in vitro* (96). Quercetin notably enhanced the expression of SR-BI in HepG2 cells in a way that depended on both concentration and time. Additionally, quercetin stimulated the binding of 1,1′-dioctadecyl-3,3,3′,3′-tetramethylindocarbocyanine perchlorate (Dil)-labeled HDL to liver cells and the uptake of 125I/3H-CE-HDL. However, using small interfering RNA (siRNA) or the specific SR-BI inhibitor, BLT-1, blocked the binding of Dil-HDL and the selective uptake of HDL-C induced by quercetin (96) (Figure 3).

Other than these common polyphenols, some other members of this family are also examined on hepatic cells for a better understanding of their effects on lipid metabolism. For instance, Baicalein which is a flavonoid extracted from Scutellaria baicalensis is administered on HepG2 cells by Jiang et al. (97). They detected that in HepG2 cells with oleic acid and palmitic acid-induced lipid accumulation, the administration of baicalein decreases the levels of TG, total cholesterol, and lipid droplets mainly through regulating SREBP1. This flavonoid lowers the levels of type 1 SREBP as well as lipogenic enzymes fatty acid synthase (FAS) and stearoyl-CoA desaturase-1 (SCD1) indirectly through pregnane X receptor (PXR) pathway (97).

#### 6.1.3 Luteolin

Luteolin is another flavonoid with the chemical formula of C15H10O6 which is extracted from Reseda luteola. A study tried to explore the capabilities of luteolin as a modulator of lipid metabolism (98). They observed that this agent is able to regulate lipid metabolism through the inhibition of SREBP-1c expression. However, they declared that this effect is indirect and luteolin affects this transcriptional factor by the means of inhibiting LXRα/β transcriptional activity (98). The results of another study on luteolin indicate that a week of dietary supplementation with this natural compound led to the increased expression of PPAR-α and its target gene, carnitine palmitoyl transferase 1 (CPT-1), in the livers of rats. luteolin-7-glucoside (L7G) also appeared to reduce the liver levels of SREBP-1, but it did not influence the protein levels of fatty acid synthase (FAS). While the mRNA levels of SREBP-2 and LDL receptor (LDLr) remained unchanged, the expression of HMGCR was diminished (99). Other studies on these polyphenols as well as some other ones including resveratrol are summarized in Table 2.

#### 6.1.4 Resveratrol

There are only a limited number of studies which investigate the effect of resveratrol and exercise on SREBP.

Jeong et al. (100) are one of the research groups which worked in this field and aimed to compare the effects of moderate exercise training versus resveratrol supplementation, both combined with a low-fat diet, on lipid metabolism in the skeletal muscle of mice with obesity induced by a high-fat diet. Thirty male C57BL/6J mice (5 weeks old) were fed a high-fat diet (45% fat) for 8 weeks to induce obesity. Subsequently, all mice switched to a low-fat diet during an 8 weeks intervention phase, where they were divided into three groups: low-fat diet control (HLC), low-fat diet with resveratrol supplementation (HLR), and low-fat diet with exercise (HLE). The HLE group performed treadmill running (30–60 min/day at 10–22 m/min, 0% incline, 5 days/week), while the HLR group received resveratrol (10 mg/kg body weight) by gavage, 5 days/week (100). They detected that mice in the HLE group showed a significant reduction in body weight, as well as decreased expression of the lipogenesis marker SREBP and the inflammatory cytokine TNF-α in skeletal muscle. In contrast, resveratrol supplementation with a low-fat diet did not produce significant changes in these parameters. Moderate aerobic exercise combined with a low-fat diet is more effective than resveratrol supplementation in improving lipid metabolism and reducing inflammation in the skeletal muscle of high-fat diet-induced obese mice (100).

Another study also aimed to compare the effectiveness of resveratrol supplementation versus aerobic exercise training, both combined with a low-fat diet, on adipogenesis and inflammation-related markers in adipose tissue of high-fat diet-induced obese mice (101). They used fifteen male C57BL/6 mice (4 weeks old) were initially fed a high-fat diet (45% fat) for 12 weeks to induce obesity, followed by a low-fat diet (10% fat) for 8 weeks. The mice were divided into three groups (*n* = 5 each): low-fat diet control (HLC), low-fat diet plus resveratrol supplementation (HLR), and low-fat diet plus aerobic exercise (HLE). The HLE group underwent treadmill running for 30–60 min per day at 10–22 m/min, 0% incline, five times per week for 8 weeks. At the end of the 20 weeks protocol, epididymal fat pads were collected for analysis (101).

The HLE group exhibited significantly lower body weight and adipose tissue mass compared to the HLC group. Fatty acid synthase (FAS) expression was significantly reduced in both HLR and HLE groups compared to HLC, while acetyl-CoA carboxylase (ACC) expression was significantly decreased only in the HLE group. Tumor necrosis factor-alpha (TNF-α) expression was significantly reduced in both HLR and HLE groups, and monocyte chemoattractant protein-1 expression was significantly lowered in the HLE group. Taken together, both resveratrol supplementation and aerobic exercise, when combined with a low-fat diet, improved adipogenesis and inflammatory markers in adipose tissue compared to diet alone. However, aerobic exercise appeared to be more effective in reducing obesity-induced metabolic dysfunction (101).

#### 6.1.5 Bergamot

Bergamot (*Citrus bergamia* Risso et Poiteau) is a small, citrus fruit primarily cultivated in the Calabria region of southern Italy. It is renowned for its essential oil, which has widespread applications in the pharmaceutical, cosmetic, and food industries. Bergamot oil is a key ingredient in Earl Gray tea and is valued for its distinctive aroma and potential therapeutic properties. Bergamot is rich in polyphenolic compounds, including flavonoids such as naringin, neoeriocitrin, neohesperidin, brutieridin, and melitidin (102). These compounds are responsible for its strong antioxidant and anti-inflammatory properties. Notably, brutieridin and melitidin exhibit statin-like activity, potentially contributing to lipid regulation through inhibition of HMG-CoA reductase (102, 103).

Bergamot flavonoids help lower lipid levels through several key mechanisms that affect lipid metabolism. They activate sirtuin-1 and AMPK-α—important regulators of cellular energy balance—which boost the breakdown of fatty acids by stimulating CPT1. At the same time, they suppress the production of very-low-density lipoprotein (VLDL) by inhibiting HNF4 and SREBP-1, two proteins involved in lipid synthesis (104). Additionally, bergamot flavonoids enhance the activity of LDL receptors in two ways: by increasing the expression of these receptors via PKC activation, and by promoting their movement to the cell surface through PPAR-γ activation. These combined effects lead to a notable decrease in LDL cholesterol and shift LDL particles from smaller, more harmful types to larger, less dangerous ones—ultimately improving the overall lipid profile (105).

Notwithstanding the beneficial effects of bergamot in regulating lipid metabolism, the studies investigating the effects of this agent on the expression of hepatic transcription factors is limited. A recent study in 2022 has investigated the health benefits of dietary fibers (DFs) extracted from bergamot, focusing on weight loss and cholesterol-lowering effects, along with the underlying biological mechanisms (106). In a 6 weeks feeding trial on Sprague-Dawley rats, bergamot DFs showed dose-dependent protective effects against metabolic syndrome. They helped reduce body weight gain, BMI, and Lee’s index without suppressing appetite. Bergamot DFs also significantly lowered triglycerides (TG), total cholesterol (TC), LDL-C, and atherogenic index (AI) in rats fed a high-fat diet and improved liver pathology. Exploring the underlying mechanisms of these effects shows that bergamot is able to decrease the expression of SREBP-1c and SREBP-2 in hepatic tissues (106).

In another study, the effect of bergamot PF extract on 2D and 3D hepatocyte cultures was also examined (107). In this study, different liver cell models were treated with bergamot PF extract to evaluate its effects under various conditions. In the 2D culture model, McA Rh-7777 rat hepatoma cells were treated for 24 h with 50 μM oleic acid conjugated to fatty acid-free bovine serum albumin (BSA) to induce lipid accumulation. Alongside oleic acid, the cells were exposed to bergamot PF extract dissolved in the culture medium at concentrations of 0.001, 0.01, 0.1, and 1 μg/mL (107). For the 3D spheroid model, composed of HepG2 and LX-2 cells, treatment began 24 h after cell seeding. These spheroids were exposed to bergamot PF extract at a concentration of 1 μg/mL for a total of 72 h, with the treatment medium being refreshed every 48 h to maintain consistent exposure. Similarly, primary human liver organoids, formed from cryopreserved hepatocytes, were treated with 1 μg/mL bergamot PF extract starting 24 h after seeding. The treatment continued for 6 days, with half of the medium replaced every 48 h to ensure the presence of fresh extract throughout the duration of the experiment (107). This integrated treatment approach across different liver models allowed for a comprehensive assessment of bergamot PF extract’s potential metabolic effects. Their results show that *in McA Rh7777*, following a 24 h incubation period, a dose-dependent rise in Srebp-1c levels was noted (*p* = 0.01). in 3D HEPG2/LX2 Spheroids, after a 96 h incubation, they found that the bergamot PF extract had no effect on the expression levels of SREBP-1C (107).

### 6.2 PPARs

#### 6.2.1 Curcumin

Curcumin is one of the polyphenols which is able to alter the expression of PPARs. An *in vitro* study in 2009 shows that CUR is able to decrease the levels of LDLR in activated hepatic satellite cells, leading to a reduction in cellular cholesterol levels. The activation of peroxisome proliferator-activated receptor-γ (PPARγ) by curcumin influenced the expression of transcription factors known as sterol regulatory element-binding proteins (SREBPs) in activated hepatic stellate cells (HSCs), ultimately suppressing the expression of the LDLR gene. A recent study attempted to show the effectiveness of fermented Curcuma longa L. in the development of alcoholic fatty liver in mice and explored the mechanisms involved. They detected that C. longa. pretreatment notably reduced the increased accumulation of hepatic lipid droplets caused by ethanol consumption. When compared to the ethanol-treated control group, mice that underwent FT pretreatment displayed a decrease in the production of cytochrome P4502E1 (CYP2E1), SREBP-1c, and acetyl-CoA carboxylase. On the other hand, they had higher levels of AMP-activated protein kinase, PPAR-α, and carnitine palmitoyltransferase 1 (CPT-1). Overall, CUR shows great potential as a liver protectant in preventing alcoholic fatty liver by influencing the processes of fatty acid production and breakdown; however, further studies need to be done to ensure long-term efficacy and safety of this agent (108).

For a better understanding of the effects of CUR on the cholesterol efflux process of adipocytes, Dong et al. (109) used Rabbit subcutaneous adipocytes and treated these cells with 5, 10, and 20 μg/ml curcumin. They assessed the levels of PPARγ mRNA in these cells after CUR treatment and found out that CUR has the ability to enhance cholesterol efflux from adipocytes by the means of increasing the expression of PPARγ in a dose-dependent manner. They confirmed these results by pre-treating these cells with GW9662 (a potent and selective PPARγ antagonist), and observed that the increased expression of PPARγ (after CUR treatment) is partially prevented (109).

#### 6.2.2 Quercetin

Quercetin is also another flavonoid which is approved to enhance conditions associated with metabolic syndrome, specifically issues like excess body weight, abnormal lipid levels, and glucose intolerance (110). In an *in vivo* study, quercetin was orally administered (10 g quercetin/kg food in a high-sucrose diet) to rats and morphometric and metabolic factors, as well as the transcriptomic profiles of the liver and retroperitoneal fat of these rats were evaluated afterward. The relative weights of epididymal and retroperitoneal fat were notably reduced in the animals treated with quercetin. Additionally, the PD-Q rats showed a smaller area under the glycemic curve and a lower fasting insulin level (110). Although there were no alterations in total cholesterol levels, the overall triglyceride levels decreased in both the serum and liver of the PD-Q rats. The transcriptomic analysis of the liver and adipose tissue supported the metabolic and structural observations, showing a pattern aligned with insulin-sensitizing changes, with key regulatory factors identified as PPARγ, Adipoq, Nos2, and Mir378 (110). Other studies on these polyphenols as well as some other ones including resveratrol are summarized in Table 3.

**TABLE 3:** Studies exploring the effects of either polyphenols, exercise, or their combination on hepatic transcriptional factor, peroxisome proliferator-activated receptors (PPARs).

Polyphenol	Model of study	Method	Route of administration	Result(s)	References
Curcumin	*In vitro*	Administering CUR on hepatic satellite cells	–	–	–
	*In vivo*	Control groups: either distilled water or 5 g/kg thanol for 8 days. Treatment groups: either 300 mg/kg milk thistle or CUR before receiving ethanol	Oral	Increasing accumulation of hepatic lipid droplets caused by ethanol through increasing PPAR-α	(108)
	*Ex vivo*	Rabbit subcutaneous adipocytes	Incubation with 5, 10 and 20 μg/ml curcumin for 24 h	Increasing cholesterol efflux through increasing the expression of PPARγ	(109)
	*In vitro*	HepG2	–	Down-regulating mRNAs of the PPARα target genes CD36/fatty acid translocase and fatty acid binding protein 1	(113)
Quercetin	*In vivo*	Adult male rats: high-sucrose diet and quercetin diet	Oral, 10 g quercetin/kg diet	Decreasing fasting insulin level and TG levels through regulating PPARγ, Adipoq, Nos2, and Mir378	(110)
	*In vitro*	HepG2 cells	–	Increasing the expression of PPARγ	(96)
	*In vitro*	Human macrophage cell line	30 μM	Up-regulating PPARγ and ABCA1	(116)
Luteolin	*In vitro*	Macrophages	–	SREBP-1c expression through decreasing LXR	(98)
	*In vivo*	Rats	–	Reducing SREBP-1 in liver	(99)

#### 6.2.3 Bergamot

The number of studies in this field are very restricted. Bergamot PF extract has shown promising effects in combating liver steatosis, although the precise mechanisms responsible for these benefits are not yet fully understood. In one study, we investigated the impact of bergamot PF extract on both 2D and 3D hepatocyte culture models, including rat cells, human hepatoma cells, and primary human hepatocytes (107). Their results demonstrated that, in 2D cultures, treatment with bergamot PF significantly reduced intracellular lipid accumulation. This reduction was accompanied by increased expression of genes involved in fatty acid β-oxidation—specifically *Acox1*, *Ppar*α, and *Ucp2*—as well as genes associated with lipophagy, such as *Atg7*. These lipid-lowering effects were further confirmed in more physiologically relevant 3D models, including hepatic spheroids and liver organoids. Overall, their findings suggest that bergamot PF extract reduces intracellular lipid storage, likely by enhancing metabolic pathways related to β-oxidation and lipid degradation, highlighting its potential therapeutic value in managing liver steatosis (107).

Another study tried bergamot on Sprague-Dawley (SD) rats for 6 weeks and found that in addition to controlling weight, bergamot DFs significantly lowered TG, TC, LDL-C, and the AI, all of which were elevated due to a high-fat diet. Liver histological analysis confirmed improvements in fat-related liver damage. At the molecular level, Western blot analysis revealed that bergamot DFs enhanced the expression of liver proteins involved in cholesterol breakdown, such as LXRα and CYP7A1, while reducing the expression of lipogenic proteins including SREBP-1c, FAS, ACC, and SREBP-2. Furthermore, gene expression analysis via qRT-PCR showed that the DFs upregulated key thermogenic and metabolic regulators—PGC-1α, PRDM16, UCP-1, and PPARγ—in brown adipose tissue, indicating enhanced energy expenditure (107).

The study also found that bergamot DFs helped normalize the gut microbiota composition disrupted by a high-fat diet. Among the different fiber types tested, soluble dietary fiber (SDF) and total dietary fiber (TDF) proved more effective in promoting weight loss and lowering lipid levels than insoluble dietary fiber (IDF) at equivalent doses. In conclusion, bergamot-derived dietary fibers exhibited strong potential to reduce body weight and blood lipid levels by modulating lipid metabolism, stimulating thermogenesis, and improving gut health. These findings support their potential use in developing functional foods aimed at preventing or managing obesity and hyperlipidemia (107).

### 6.3 RAR/RXR and FXR/LXR

Studies on these transcriptional factors are very limited and curcumin, quercetin, Baicalein, and resveratrol are the only polyphenols on which studies are conducted.

#### 6.3.1 Curcumin

Yan et al. (111) are one of the research groups which tried to examine the effects of curcumin on these transcriptional factors. They examined the impact of curcumin on the interplay between the metabolism of endogenous bile acids and the metabolism of external xenobiotics in C57BL/6 mice with NAFLD caused by a high-fat and high-fructose diet (HFHFr) (111). Their findings show that curcumin treatment significantly reduced hepatic steatosis and normalized serum biochemical parameters in mice on a high-fat-high-fructose diet. Curcumin successfully adjusted the expression levels of CYP3A and CYP7A in the fatty liver condition, thereby restoring metabolic function. Additionally, curcumin regulated lipid synthesis, as shown by the changes in the expressions of CD36, SREBP-1c, and FAS. Moreover, there was a notable decrease in the expressions of FXR, SHP, and Nrf2 in the HFHFr-fed mice (111). They also observed that Prior treatment with the LXRα antagonist GGPP reduced the impact of curcumin on CYP3A, CYP7A, and SREBP-1c (111). In an *ex vivo* study, Dong et al. (109) tried to explain the underlying mechanisms by which CUR is able to increase cholesterol efflux from adipocytes. They used RT-PCR for detecting the altered gene expression caused by CUR in these cells and found out that LXR is one of the main genes which is up-regulated as a result of CUR treatment (109). A similar study also used CUR on THP-1 Macrophage-Derived Foam Cells to assess the same effects (112). They observed that CUR significantly enhanced the expression of ATP-binding cassette transporter 1 (ABCA1), facilitated the removal of cholesterol from foam cells derived from THP-1 macrophages, and lowered cholesterol levels within cells. It also stimulated AMP-activated protein kinase (AMPK) and SIRT1, which subsequently activated LXRα in these foam cells (112). Blocking AMPK/SIRT1 activity with a specific inhibitor or small interfering RNA may prevent LXRα activation and eliminate the curcumin-induced expression of ABCA1 and cholesterol efflux. Consequently, curcumin promoted cholesterol efflux by increasing ABCA1 expression via the activation of the AMPK-SIRT1-LXRα signaling pathway in foam cells derived from THP-1 macrophages (112). Peschel et al. (113) tried CUR on HepG2 cells and found out that this method leads to a concentration-dependent increase of up to seven times in LDL-receptor mRNA levels. In contrast, the mRNAs for the genes responsible for the sterol biosynthetic enzymes HMG CoA reductase and farnesyl diphosphate synthase show only a slight increase at high curcumin concentrations, which also results in reduced cell viability (113).

In another point of view, CUR is also effective for enhancing cognitive function in mice which have developed dementia as a result of lipid metabolic disorder (114). In this regard, CUR enhanced the unique cognitive and memory functions in transgenic mice with Alzheimer’s Disease. In these mice, the total serum cholesterol levels decreased with a curcumin-rich diet, while high-density lipoprotein levels increased. This dietary intake of curcumin was linked to lower levels of Aβ and higher levels of liver X receptor-β, ATP-binding cassette A1, and apolipoprotein A1 in the CA1 region of the hippocampus (114). In the brains of Alzheimer’s Disease mice that were given a curcumin diet, the levels of mRNA and protein for retinoid X receptor-α, liver X receptor-β, and ATP binding cassette A1 were increased (114).

#### 6.3.2 Quercetin

Quercetin is also another polyphenol which has some effects on the expression levels of these transcriptional factors. *In vitro* studies has shown that quercetin is able to increase the expression of LXR in hepatic cells and this effect can be reverted by the administration of LXR siRNA (96). Other than LXR, quercetin is also able to impact RXR. According to a study by Jiang et al. (97) which cultured human adipocytes either in a state where they are actively producing and gathering more lipids through lipogenesis (“active lipogenic state”) or in a state where they are keeping their stored lipids stable without any increase (“lipid storage state”); it is reported that In the “lipid storage state,” a prolonged treatment involving three doses over 72 h with low levels of quercetin and ferulic acid together led to a significant reduction in stored lipid levels, changes in lipid composition, and modulation of genes associated with lipid metabolism. This suggests a notable involvement of PPARα and RXRα (115). However, In the “continuous lipogenic state,” the impacts of quercetin and ferulic acid differ significantly, showing minimal alterations in gene expression and lipid makeup. Additionally, there is no identifiable role of PPARα/RXRα, and a concentration ten times greater is needed to reduce stored lipid levels (115). Plus, Quercetin, even at concentrations of up to 30 μM, did not harm differentiated THP-1 cells. It enhanced the messenger RNA and protein levels of ABCA1 in these cells, with the most significant effects observed at 0.3 μM after 4–8 h of incubation. Additionally, quercetin raised the protein levels of PPARγ and LXRα within just 2 h of treatment. Since PPARγ and LXRα play crucial roles as transcription factors for ABCA1, the increase in ABCA1 caused by quercetin may be mediated through these factors (116).

#### 6.3.3 Resveratrol

Resveratrol is also an important polyphenol which is effective on LXR and FXR either administered alone or in combination with exercise. A study evaluated the therapeutic effects of resveratrol, exercise training, and their combination on hepatic function and gene expression in rats with NAFLD (117). The animals were randomly assigned to seven groups, including controls and interventions involving resveratrol, interval or continuous exercise, and their combinations. The control group exhibited significantly higher mRNA expression levels of LXR, FXR, and SIRT1 compared to all other experimental groups (*p* < 0.001) (117). Additionally, both the resveratrol-treated and exercise-trained groups demonstrated elevated expression of these genes relative to the patient and saline groups (*p* < 0.01). Notably, the combined treatment of resveratrol and exercise training resulted in a markedly greater increase in the mRNA expression of FXR, LXR, and SIRT1 compared to either intervention alone (*p* < 0.001) (117). Their results demonstrated that both resveratrol and exercise independently enhanced the expression of Sirt1, Lxr, and Fxr, while also reducing markers of liver injury and apoptosis. However, the combined intervention of resveratrol with either interval or continuous exercise yielded significantly greater improvements across all parameters. These findings suggest a synergistic effect between resveratrol and physical activity, offering a promising combined approach for managing NAFLD through improved liver function and regulation of transcriptional pathways involved in lipid metabolism (117).

Similarly, another study also investigated the effects of resveratrol on SIRT1 and other genes related to lipid metabolism (118). In this study, 28 male rats (weighing 260 ± 10 g, 8 weeks old) were randomly assigned to four groups (*n* = 8 per group): control (C), aerobic training (T), supplement (S), and combined training with supplement (T-R). The training groups underwent aerobic exercise on a treadmill for 14 weeks (five sessions per week, 45 min per session) (118). Tissue protein levels of UCP-1, SIRT1, and PGC-1α were measured using ELISA. Data were analyzed using ANOVA, with statistical significance set at *p* ≤ 0.05. Significant increases in SIRT1 and PGC-1α were observed in the liver and subcutaneous/visceral adipose tissues, respectively (*P* ≤ 0.05 and *P* ≤ 0.001). UCP-1 levels were significantly higher in the group receiving both resveratrol supplementation and aerobic training (*P* ≤ 0.001) (118). The findings suggest that combining resveratrol supplementation with aerobic exercise produces greater effects on increasing UCP-1, SIRT1, and PGC-1α protein expression than either intervention alone, potentially improving liver function and promoting the conversion of subcutaneous white adipose tissue to a beige or intermediate phenotype (118).

In another study, 32 male mice (average weight 16 ± 4 g) were randomly assigned to four groups (*n* = 8): Control, resveratrol, Aerobic Exercise (Exe), and Resveratrol combined with Aerobic Exercise (resveratrol + Exe) (119). Mice in the resveratrol and resveratrol + Exe groups received resveratrol via gavage five times per week for 8 weeks. The Exe and resveratrol + Exe groups underwent treadmill training at 50%–65% of VO_2_max, five sessions per week for 8 weeks. They observed that resveratrol alone increased FNDC5, SIRT1, and UCP1 gene expression compared to the control group (119). Aerobic exercise alone significantly upregulated all five genes compared to control. Notably, the combined resveratrol + Exe group showed significantly higher expression of all thermogenic and browning-related genes compared to either intervention alone. Additionally, the resveratrol + Exe group exhibited improved lipid profiles, with higher HDL and lower LDL, triglycerides, and total cholesterol (119).

Zahedi et al. (120) also identified that resveratrol supplementation combined with aerobic exercise likely exerts a stronger effect on increasing SIRT1 and PGC-1α expression in the soleus muscle and inguinal adipose tissue, as well as UCP-1 expression in inguinal adipose tissue, compared to either intervention alone. This combined approach appears to be more effective than resveratrol or physical activity alone in promoting the browning of subcutaneous white adipose tissue, potentially inducing a shift toward a beige or intermediate adipocyte phenotype (120).

There is also one paradoxical study in this field that aimed to evaluate the effects of aerobic exercise and resveratrol supplementation on mitochondrial biogenesis in the skeletal muscle of mice with obesity induced by a high-fat diet (121). Forty male C57BL/6 mice (4 weeks old) were randomly assigned to four groups (*n* = 10 per group): normal diet (NC), high-fat diet (HC), high-fat diet with resveratrol supplementation (HRe), and high-fat diet with aerobic exercise (HE) (121). The aerobic exercise regimen involved treadmill running for 40–60 min per day at a speed of 10–14 m/min, 0% incline, 4 days per week for 16 weeks. Resveratrol was administered orally at a dose of 25 mg/kg body weight, four times per week for the same duration.

Results showed a significant reduction in COX-IV mRNA expression in the HC group compared to the NC group (*p* < 0.05), indicating a negative impact of the high-fat diet. However, the HE group exhibited significantly increased expression of SIRT3, PGC-1α, and COX-IV mRNA in skeletal muscle compared to both the HC and HRe groups (*p* < 0.05) (121). In conclusion, high-fat diet-induced obesity suppressed mitochondrial biogenesis gene expression in skeletal muscle, while aerobic exercise effectively restored and enhanced it. Resveratrol supplementation alone did not significantly influence mitochondrial biogenesis, suggesting that aerobic exercise is the more effective intervention for improving mitochondrial function in obese skeletal muscle (121).

#### 6.3.4 Baicalein

Other than these polyphenols, Baicalein which is a flavonoid derived from Scutellaria baicalensis is also examined on HepG2 cells (97). According to this study, this polyphenol is able to enable the movement of pregnane X receptor (PXR) into the nucleus and regulate the expression of its target genes. In HepG2 cells that experienced lipid buildup due to oleic acid and palmitic acid, treatment with baicalein decreased the levels of triglycerides, total cholesterol, and lipid droplets (97). The findings from the computer simulation showed that baicalein acts as a natural ligand for PXR (97). Luetolin is also another important member of the flavonoid family which has the ability to affect LXRα/β (98). An *in vitro* study examined the expression levels of LXR-target genes using real-time RT-PCR. Specifically, they looked at sterol regulatory element binding protein 1c (SREBP-1c) in liver cells and ATP-binding cassette transporter (ABC)A1 in macrophages. The lipid levels in liver cells were assessed through Oil Red staining (98). The findings of this revealed, for the first time, that luteolin effectively blocked the transcriptional activity induced by LXRα/β agonists. As a result, it inhibited SREBP-1c expression, reduced lipid accumulation, and affected ABCA1 expression. Thus, luteolin has the potential to counteract hypertriglyceridemia linked to LXR activation, suggesting possible therapeutic benefits for related conditions (98). Other studies on these polyphenols as well as some other ones including resveratrol are summarized in Table 4.

**TABLE 4:** Studies exploring the effects of either polyphenols, exercise, or their combination on liver X receptors (LXR), farnesoid X receptor (FXR), retinoid X receptor (RXR), and retinoic acid receptor (RAR).

Polyphenol	Transcriptional factor	Model of study	Method	Result(s)	References
Curcumin	FXR and LXR	*In vivo*	C57BL/6 mice of non-alcoholic fatty liver disease induced by high-fat and high-fructose diet	Increasing FXR and decreasing LXR and restoring metabolism capability	(111)
	LXR	*Ex vivo*	Rabbit subcutaneous adipocytes	Increasing cholesterol efflux through increasing the expression of LXRα	(109)
	LXR	*In vitro*	HepG2	LXRα expression and accumulation of mRNA of the LXRα target gene ABCg1 were increased at low curcumin concentrations	(113)
	LXR and RXR	*Iin vivo*	APP/PS1 double transgenic mice: dementia group, low-dose, and high-dose groups	Increasing LXR and RXR expression in brains of mice with dementia	(114)
	LXR	*In vitro*	THP-1 macrophage-derived foam cells	Increasing cholesterol efflux by upregulating ABCA1 expression through activating AMPK-SIRT1-LXRα signaling	(112)
Quercetin	LXR	*In vitro*	HepG2 cells	Increasing the expression of LXR	(96)
	LXR	*In vitro*	Human macrophage cell line	Up-regulating LXRα and ABCA1	(116)
Baicalein	PXR	*In vitro*	HepG2 cells	Decreasing the levels of triglycerides, total cholesterol, and lipid droplets through PXR regulation	(97)
Luteolin	LXR	*In vitro*	Macrophages	Abrogating LXRα/β agonist-induced LXRα/β transcriptional activity	(98)
Resveratrol	LXR	*In vivo*	Mice	Increased the bile acid pool size but did not affect cholesterol consumption or *de novo* cholesterol synthesis through decreasing LXRα	(125)
	LXR	*In vitro*	Human macrophages	Increasing LXR-α and ABCA1 and ABCG1	(126)

## 7 Limitations of research

Polyphenol formulations discussed in this review are developed with varying amounts and different ingredients that impact their bioavailability, thus making it complicated to suggest a single optimal dose. However, formulation-specific doses can be suggested based on the scientific research for each product. Most of the studies discussed in this review recruited young healthy participants and there are no data available for the effect of polyphenols on older adults. In addition, research on the chronic effects of polyphenol consumption based on specific formulations, are required to evaluate their effects on recovery from lipid metabolism disorders.

Curcumin has garnered much attention due to its potential therapeutic effects, However, one of its main limitations is its poor bioavailability, meaning that very little of it is absorbed in the gastrointestinal tract when taken orally. This issue significantly limits its therapeutic potential because the body can’t effectively utilize curcumin in its natural form (122). Curcumin is not easily absorbed by the body due to its hydrophobic nature and rapid metabolism in the liver. When taken orally, a large proportion of it is metabolized before it can reach the bloodstream and exert its effects. While studies have used doses ranging from 500 mg to 12 g per day, typical daily doses in supplement form are much lower, usually around 500–1,000 mg of curcumin. This could be more effective when combined with absorption-enhancing agents like piperine or fat.

In general, curcumin should be used cautiously in high doses, and the ideal dose should be individualized based on the specific therapeutic goals and the use of adjuncts to improve bioavailability. Further human studies in this field are required for coming to a conclusion about the dosage and side effect of this agent (122).

## 8 Concluding remarks

The rising incidence of metabolic diseases such as obesity, type 2 diabetes, dyslipidemia, and NAFLD underscores the urgent need for effective preventive and therapeutic strategies. This review has systematically explored the role of dietary polyphenols—particularly curcumin, quercetin, baicalein, luteolin, and resveratrol—and their interaction with hepatic transcriptional regulators, including SREBP-1c, PPARα, PPARγ, LXR, FXR, RXR, and RAR. These transcription factors are central to lipid metabolism, influencing lipid synthesis, oxidation, transport, and storage within hepatocytes.

Our findings indicate that polyphenols exhibit potent regulatory effects on these transcriptional networks, acting through both direct modulation and indirect signaling cascades such as AMPK, SIRT1, and PXR. Among the polyphenols studied, curcumin and resveratrol show particularly strong potential in attenuating lipogenesis and promoting lipid oxidation. Notably, these effects are often context- and dose-dependent. In some studies, curcumin upregulates PPARα expression, while in others, it exerts an inhibitory effect—likely due to variations in experimental design, dosage, duration of treatment, and model system. This reinforces the complexity of polyphenol action and the need for standardized experimental approaches in future research.

Exercise is another critical modulator of hepatic lipid metabolism, and our analysis demonstrates that physical activity—especially aerobic and high-intensity interval training (HIIT)—enhances the expression and activation of lipid-regulating transcription factors, notably PPARγ and LXRα. The synergistic potential of combining exercise with polyphenol supplementation was also highlighted, with several studies reporting additive or even multiplicative effects in improving lipid profiles, insulin sensitivity, and reducing hepatic steatosis. These combined interventions not only regulate hepatic lipid metabolism more effectively but may also offer enhanced protection against cardiovascular and metabolic complications.

Importantly, our review identifies key gaps in the current literature. There is a lack of longitudinal, dose-optimized, and clinically controlled studies that evaluate the combined impact of polyphenol intake and exercise on hepatic transcriptional regulation in human populations. Additionally, the overlapping and sometimes contradictory effects of polyphenols on the same transcription factor—such as SREBP-1c or PPARα—highlight the need for deeper mechanistic investigations, particularly at the epigenetic and post-translational levels.

In summary, the integrated use of dietary polyphenols and exercise represents a promising and multifaceted approach to managing hepatic lipid metabolism and mitigating the risk of metabolic disorders. By targeting key transcriptional regulators, these interventions offer a potential non-pharmacological strategy for early prevention and adjunctive treatment of metabolic diseases. Further high-quality, standardized, and mechanistically detailed studies—especially in human subjects—are warranted to translate these findings into practical clinical applications. It is important to acknowledge that the regulatory effects of polyphenols on hepatic transcriptional factors are not always consistent across studies. For instance, curcumin has been reported to both increase and decrease the expression of PPARα depending on the experimental context. Such discrepancies are likely influenced by several factors, including the dosage and duration of curcumin administration, route of delivery, experimental model (e.g., cell line, animal species, or tissue type), and the metabolic or disease state of the system studied. Furthermore, polyphenols often act through multiple, overlapping transcriptional regulators, and may influence one factor indirectly via modulation of another (e.g., curcumin’s activation of PPARγ may impact SREBP or LXR signaling). These overlapping pathways and context-dependent effects highlight the complexity of interpreting polyphenol action and underscore the need for standardized, dose-controlled studies to clarify their precise mechanisms in regulating lipid metabolism.
